# Direct Comparison of the Hinge-Cleaving Proteases IgdE and BdpK for LC-MS-Based IgG1 Clonal Profiling

**DOI:** 10.1021/acs.analchem.3c03712

**Published:** 2023-12-18

**Authors:** Danique M. H. van Rijswijck, Albert Bondt, Naomi de Kat, Rolf Lood, Albert J. R. Heck

**Affiliations:** † Biomolecular Mass Spectrometry and Proteomics, Bijvoet Center for Biomolecular Research and Utrecht Institute for Pharmaceutical Sciences, University of Utrecht, Padualaan 8, Utrecht 3584 CH, The Netherlands; ‡ Netherlands Proteomics Center, Padualaan 8, Utrecht 3584 CH, The Netherlands; § Genovis AB, Scheelevägen 2, 223 63 Lund, Sweden

## Abstract

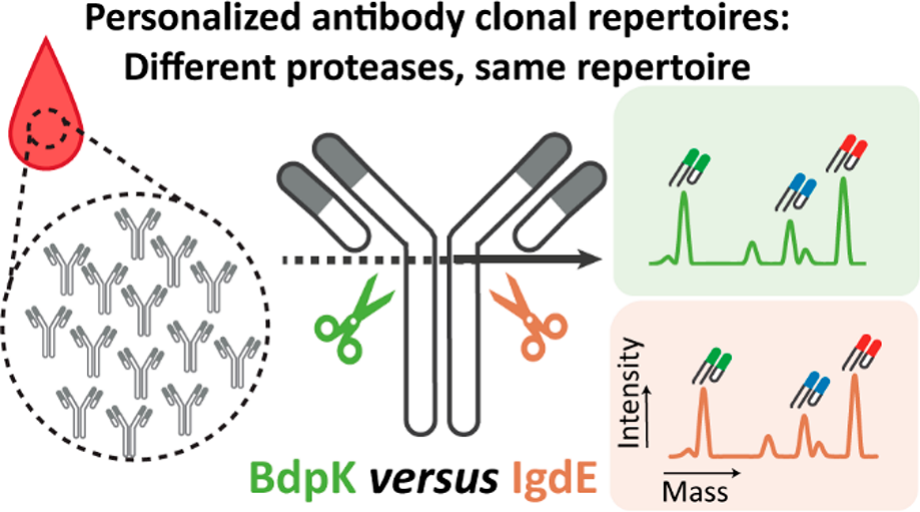

Human antibodies are heterogeneous molecules primarily due to clonal sequence variations. Analytical techniques to assess antibody levels quantitatively, such as ELISA, lack the power to resolve abundances at the clonal level. Recently, we introduced an LC-MS-based approach that can distinguish and quantify antibody clones using the mass and retention time of their corresponding Fab-fragments. We used specific hinge-cleaving protease IgdE (FabALACTICA) to release the Fab-fragments from the constant Fc region of the antibody. Here, we explore an alternative IgG1 hinge-cleaving protease, BdpK (FabDELLO), and compare it directly to IgdE for use in IgG1 repertoire profiling. We used IgdE and BdpK in parallel to digest all IgG1s from the same set of plasma samples. Both proteases cleave IgG1 specifically in the hinge, albeit via different mechanisms and at two distinct cleavage sites. Notwithstanding these differences, the Fab fragments generated by IgdE or BdpK produced highly similar clonal repertoires. However, IgdE required ∼16 h of incubation to digest plasma IgG1s, while BdpK required ∼2 h. We authenticated the similarity of the clones by top-down proteomics using electron transfer dissociation. We conclude that BdpK performs very well in digesting polyclonal plasma IgG1s and that neither BdpK nor IgdE displays detectable biases in cleaving IgG1s. We anticipate that BdpK may emerge as the preferred protease for IgG1 hinge-digestion because it offers a shorter digestion time compared to IgdE, an equally specific digestion site, and no bias against any IgG1 present in plasma.

## Introduction

An important part of the adaptive immune system is represented by B cells. They express a unique B cell receptor (BCR) that recognizes and binds to antigens in a very specific manner. The uniqueness and specificity of these BCRs originates from the way they are formed, namely by the somatic recombination of V-, D-, J-, and C-germline segments.^1,2^ This recombination of segments can, in theory, generate more than 10^13^ possible BCRs, with each targeting a specific antigen.^3,4^ Once an antigen is bound to its cognate BCR, the B cell is activated and starts to proliferate and produce antibodies. The produced antibodies are secreted and spread throughout the body to target specific antigens.^2^ Techniques that are available to investigate the B cell response can focus on either the BCRs, e.g., BCR sequencing techniques at the DNA or RNA level,^4,5^ or on the generated antibodies, e.g., by using ELISA-based techniques. Although ELISA-based techniques can give insight into the total quantity of antibodies, sometimes even with isotype resolution, these assays cannot resolve antibodies at the clonal level.^6^ Distinguishing between antibodies at the clonal level is challenging due to the vast array of antibody sequence variants and the added-on variability induced by N- or O-glycosylation present on the Fc part of the antibodies. Notwithstanding these challenges, we need to distinguish antibodies at the clonal level to better understand the immune system and find new potential candidates for monoclonal antibody (mAb) therapy development. To fill this gap, techniques that can focus at the protein level on the antibody repertoires, e.g., Ig-seq^4,7^ and liquid chromatography mass spectrometry (LC-MS)-based antibody clonal profiling,^8,9^ are essential.

We recently introduced an LC-MS-based method to profile antibody clonal repertoires qualitatively and quantitatively. For antibody clonal profiling, we remove the constant Fc part from the antibodies by using specific antibody hinge-cleaving proteases. Removing the Fc part isolates the variable Fab-fragments and makes the analyte molecules simpler to analyze by eliminating the Fc glycosylation sites. The resulting intact Fab molecules, which contain all six CDRs from the light and heavy chain, can then be analyzed using an LC-MS-based approach that distinguishes unique clones by their mass and retention time.^8^

The antibody clonal profiling method relies heavily on the antibody hinge-cleaving protease. Such a protease should be very specific and should not exhibit a bias in cleaving certain antibodies better than others. Several bacteria produce proteases that cleave IgG’s as an evasion tactic against host immunity, and several of these have now been described, characterized, and used for applications in biotechnology, biopharma, and middle-down proteomics.^10^ IgdE is such a specific IgG1 hinge-cleaving protease that is often used and which we also used in the clonal profiling method initially.^8,11^ IgdE is a cysteine protease that is derived from the pathogen *Streptococcus agalactiae*. This protease cleaves uniquely human IgG1s at one specific site just above the hinge region, namely, at KSCDKT/HTCPPC. For activity IgdE does not need any reducing conditions or cofactors. While IgdE is very specific, it has not yet been demonstrated that it cleaves equally efficiently (without bias) for all IgG1 clones in circulation.

Recently, another protease was described: BdpK that can also cleave IgG1 in its hinge-region. BdpK is a serine protease that is derived from the nonpathogenic bacteria *Bdellovibrio bacteriovorus*. This bacterium is harmless to humans but predates other bacteria by its high enzyme-to-chromosome ratio that enables them to hydrolyze most macromolecules from other bacteria.^12^ Although *B. bacteriovorus* is harmless to humans, BdpK is a broad-acting protease. Among these substrates, it cleaves human IgG1, at one specific site, also just above the hinge region, namely, at KSCDK/THTCPPCP. The cleavage site targeted by BdpK is therefore one Threonine amino acid closer to the N-terminus of IgG1 compared to IgdE. BdpK also does not need any reducing conditions but does need calcium ions as cofactor. The specificity of BdpK arises from the tertiary structure of IgG and the exposure of only one single lysine residue located in the hinge region of IgG1 and hence is specific. This specificity is similar to broad-acting proteases, like KGP or trypsin, but different than other broad-acting proteases that are known to digest IgG1 above the hinge region, like papain.^13^ Papain is clearly less specific in digesting human IgG1, as it cleaves the molecule at different sites, rendering papain less suitable for LC-MS-based IgG1 Fab clonal profiling.^14^

Here, we directly compared and evaluated the performance of IgdE and BdpK for plasma IgG1 repertoire profiling by LC-MS. This is the first report that describes BdpK for the digestion of polyclonal plasma IgG1s and that describes the possible biases introduced by either IgdE or BdpK. Our data reveal that BdpK can digest polyclonal plasma IgG1 specifically and efficiently and that neither BdpK nor IgdE introduces detectable biases in the clonal repertoire profiles. This latter feature is important for the unbiased qualitative and, especially, quantitative profiling of antibody repertoires.

## Methods

### Plasma IgG Purification and Fab Generation Using IgdE and BdpK

We first performed experiments to test the performance of the hinge-cleaving proteases IgdE (FabALACTICA, Genovis AB, Lund, Sweden) and BdpK (FabDELLO, Genovis AB, Lund, Sweden) for Fab clonal profiling. The experiments followed procedures described previously^8^ and used buffers and conditions optimized for each protease following the vendors’ information. All experiments were conducted on donor plasma from the same source. For more details about the healthy donor plasma used, see Experimental S1 in the Supporting Information. For IgdE we used a phosphate buffer (PB) of 150 mM (pH 7), and for BdpK we used tris buffered saline (TBS) with 10 mM CaCl_2_ (pH 7.6) throughout the entire protocol. IgG was purified from plasma by using 20 μL of CaptureSelect FcXL affinity matrix slurry (Thermo Fisher Scientific), added to Pierce Spin Columns (Thermo Fisher Scientific). The affinity matrix was washed three times by using the preferred buffer for the corresponding protease. For each washing step the liquid was removed by centrifugation for 1 min at 500*g*, at room temperature. Then, we added 10 μL of plasma, together with 150 μL of the corresponding buffer, to every column. The samples were subsequently incubated under shaking conditions for 1 h at room temperature. After incubation, the flowthrough was collected, and the affinity matrix with bound IgGs was washed 4 times using 200 μL of the corresponding buffer. Finally, for the IgdE-treated plasma samples, we added 50 μL of PB, 150 mM (pH 7) containing 50 arbitrary units of IgdE (FabALACTICA, Genovis AB, Lund, Sweden). For the BdpK treated plasma samples, we added 50 μL TBS buffer with 10 mM CaCl_2_ containing 50 arbitrary units BdpK (FabDELLO, Genovis AB, Lund, Sweden). The IgdE treated samples were incubated on a thermal shaker at 37 °C for 16 h, and the BdpK-treated samples were incubated at 37 °C for 2 h. After incubation with either IgdE or BdpK, the flowthrough containing the Fab fragments generated from the bound IgG1s was collected by centrifugation for 1 min at 500*g*. Next, to analyze and profile the released Fab fragments, we employed a reversed-phase liquid chromatography coupled mass spectrometry (LC-MS) and data processing method, as previously described.^8,9^ Details about the LC-MS experiment and subsequent data processing method are described in Experimental S1 in the Supporting Information.

## Results and Discussion

### Experimental Design

**Figure 1:**
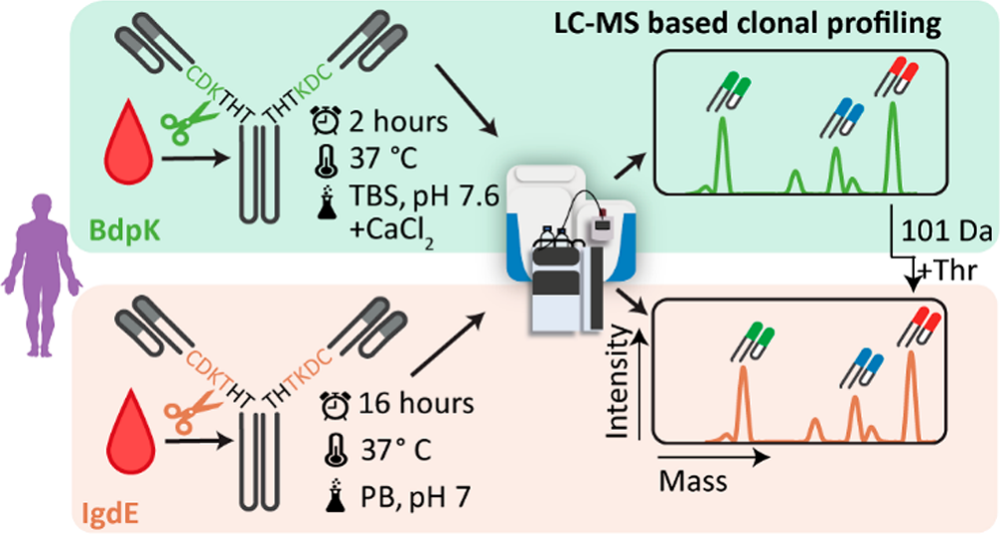
Experimental design for the comparison of the performance of BdpK (green box) and IgdE (orange box) for LC-MS-based IgG1 Fab clonal profiling. We used both proteases on the same plasma sample using the optimized buffer conditions per protease. In the LC-MS-based IgG1 clonal repertoire profiling, we define unique clones by their mass and retention time by the proteases formed Fab fragments. The intensities of the LC-MS peaks are a direct indication of the abundance of each detected clone. The masses of Fab fragments originating from the same plasma IgG1 clone digested by either BdpK or IgdE will always be 101 Da in mass apart due to the distinct cleavage sites.

To chart and monitor plasma IgG1 repertoires qualitatively and quantitatively, we recently described a mass spectrometry-based approach that can be used to distinguish antibodies at the clonal level. In this approach, we used the well-characterized IgG1 hinge-cleaving protease IgdE to cleave off and analyze the IgG1 Fab fragments. At that time, IgdE was the only known protease that was fully specific in cleaving IgG1 above the hinge region without the need of denaturing or reducing conditions. As we did not have a clear benchmark, we could not exclude whether IgdE may exhibit biases, cleaving some IgG1 molecules better than others, which could affect the quantitative aspect of the clonal profile. Recently, the second IgG1 cleaving protease BdpK was discovered. BdpK is a broad-acting protease that also cleaves the IgG1s very specifically above the hinge region. IgdE and BdpK have adjacent albeit distinct cleavage sites, whereby BdpK cleaves one Threonine amino acid closer to the N-terminus of IgG1 compared to IgdE. Here, we set out to evaluate whether also BdpK can be used for plasma IgG1 clonal profiling, benchmarking it directly versus IgdE. Theoretically, when both proteases behave the same for plasma IgG1 clonal profiling, we would expect identical clonal profiles independent of which protease is used. The sole difference would be that all Fab masses observed following BdpK digestion should be 101 Da lower in mass due to the different but adjacent digestion sites of the two used proteases (Figure 1).

### BdpK and IgdE Display Highly Similar Fab Clonal Repertoires

**Figure 2:**
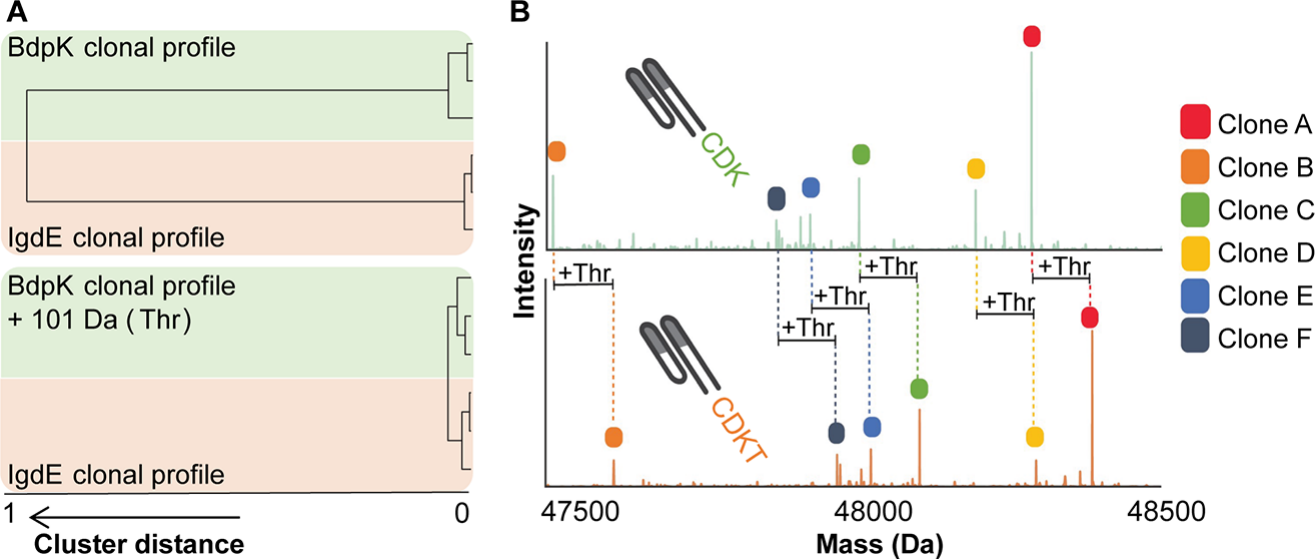
Comparison of the LC-MS-based IgG1 Fab clonal profiles generated by BdpK and IgdE. (A) Hierarchical clustering of the 6 IgG1-Fab mass plots generated from the Fab clonal repertoires of plasma from one donor collected at 3 time points digested by either IgdE or BdpK. The clustering is based on the correlation distance, before (top) and after adjusting for a mass increase of 101 Da in BdpK-generated Fab clones (bottom). (B) Deconvoluted mass plots of the same plasma sample digested with BdpK (top trace, green) or IgdE (bottom trace, orange). The colored clones represent the top 6 clones in both digests, with a 101 Da difference corresponding to a Threonine (Thr) difference in the digestion site. All plasma samples exhibited similar profiles, independent of the protease used (see also Figure S1).

We acquired 6 distinct Fab-based LC-MS clonal profiles: 3 were generated by using IgdE, and the other 3 by using BdpK. For both IgdE and BdpK digestion, the same 3 plasma samples were used which were taken at different time points from the same healthy donor. We compared the mass plots of the clonal profiles present after IgdE digestion with each other and with the mass plots of the clonal profiles present after BdpK digestion performing hierarchical clustering (Figure 2). The clonal profiles of the same donor at 3 different time points, generated by using the same protease, correlate quite well. This is in line with our earlier reported data which revealed that, in healthy donors, clonal profiles are qualitatively and quantitatively relatively stable over a time window of several months.^8^ When naively adopting the clustering to compare the clonal profiles generated by either IgdE or BdpK we observed that these did not cluster at all (cluster distance = 1). We next increased the masses of all the Fab clones present after BdpK digestion by 101 Da to account for the extra Threonine amino acid residue present on the C-terminus of the clones digested with IgdE. After this correction the mass plots of all 6 profiles cluster well together with a cluster distance <0.05 (Figure 2A). Thus, the clones present after both digestions are quantitatively and qualitatively very alike (Figures 2, S1, and S2).

### Efficiency of BdpK Seems to Be Higher

Overall, BdpK digestion yielded a higher number of detectable clones, namely, an average of ∼700 clones per analyzed plasma sample, whereas after IgdE digestion an average of ∼350 clones could be detected. Also, the total sum (i.e., total ion current) of all clonal intensities in the IgG1 repertoires was higher for the clones generated by BdpK digestion when compared to IgdE digestion, ∼ 3.7 × 10^10^ versus ∼2.4 × 10^10^ sum intensity, respectively.

These higher numbers of clones and higher total sum of all clonal intensities originated primarily from low-abundant clones that could be detected after BdpK digestion but were not detected (i.e., below threshold) after IgdE digestion (Figure S2). We compared the intensities of the top-200 overlapping clones detected in the IgG1 repertoires obtained after either digest, and we observed that they matched very well (Figure S2A). When comparing the intensities of all clones detected after either digest, thus also including the lower abundant clones, we observed that they still matched very well with an overall correlation (*R*^2^) of ∼0.72. We also compared the intensities of the overlapping clones detected in technical replicates (using the same protease), which revealed a correlation ∼0.9. This high correlation indicates that digestions by either IgdE or BdpK are very robust and reproducible (Figure S2B) and that the obtained IgG1 Fab profiles correlate very well not only qualitatively but also quantitatively.

### Validation of Shared Clonality by Top-Down Fragmentation of Selected Clones

**Figure 3:**
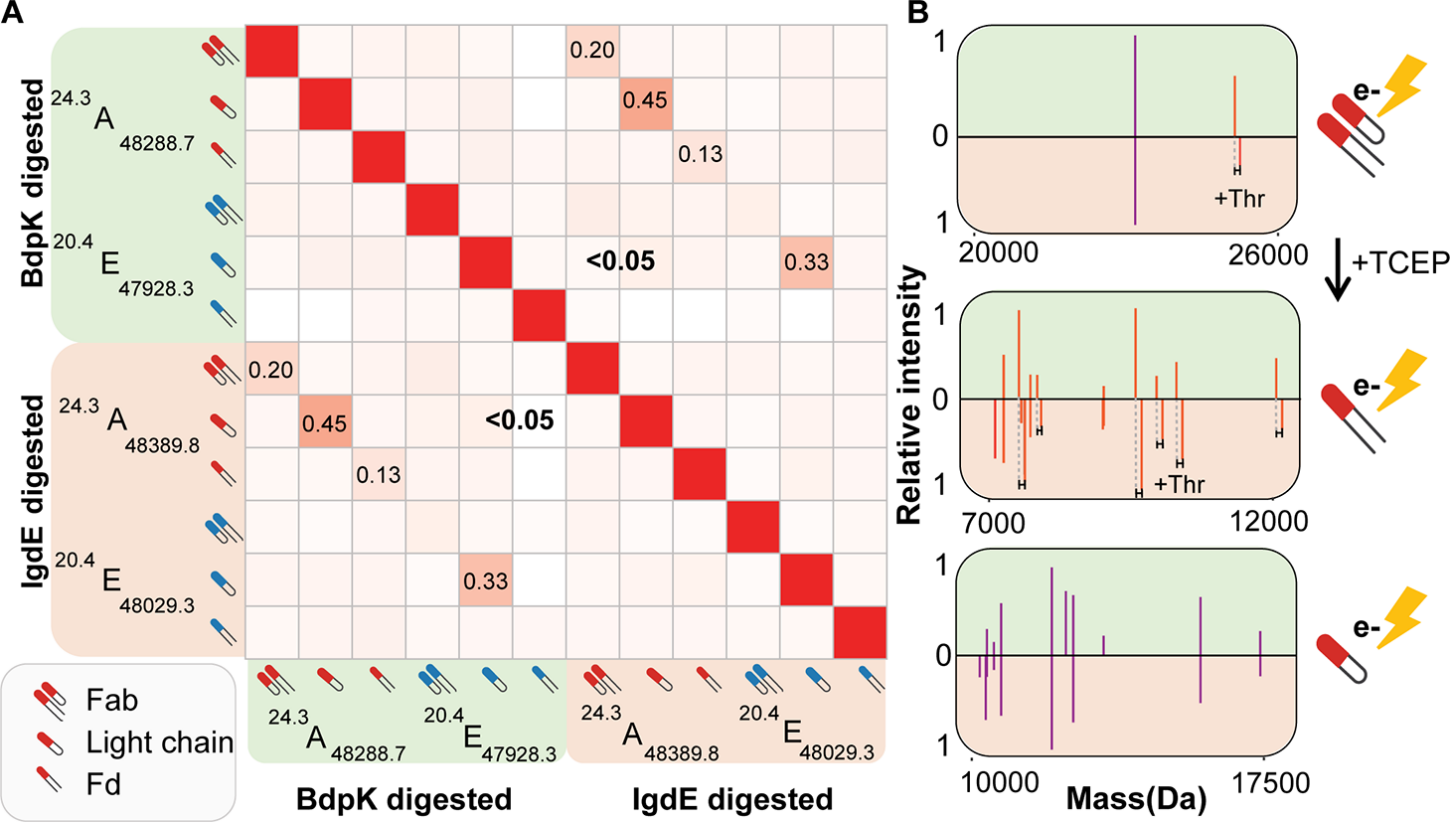
Confirmation of shared identity between clones in the IgdE or BdpK digest through top-down ETD-based analysis. (A) Heatmap depicting Pearson correlation values (ranging from 0 to 1) of raw top-down ETD MS/MS spectra for Fab clones A and E (shown in Figure 2) and for the reduced Fab clones A and E, generated by either IgdE or BdpK digestion. (B) Mirrored deconvoluted top-down ETD MS/MS spectra of clone A generated by either BdpK (green, top spectrum in the mirror plots) or IgdE (orange, bottom spectrum in the mirror plots). These mirrored deconvoluted top-down ETD MS/MS spectra are shown for intact Fabs (top spectrum) and for the reduced Fab, Lc, and Fd chains (2 bottom spectra). The middle spectrum shows the Fd fragment ions, and the bottom spectrum shows the LC fragment ions.

We aimed to find additional evidence to confirm that the shared Fab clones between IgdE and BdpK digestions are indeed identical. Therefore, we performed top-down MS/MS analysis, using electron transfer dissociation (ETD), on several abundant “shared” Fab clones. Conducting ETD on the intact Fab molecules mainly caused the inter disulfide bridge between the light chain (Lc) and N-terminal parts of the heavy chain (Fd) moieties to break, which allowed a comparison of the masses of the Lc and the Fd for Fab clones produced by either IgdE or BdpK. While the Lc masses of these shared clones were as expected the same, the Fd masses were found to be always shifted by 101 Da, corresponding to the extra Threonine (Thr) present on the C-terminus following IgdE digestion (Figure 3).

Next, we conducted ETD on the Lc and Fd separately, which we generated by first reducing the Fab clones using TCEP. This reduction of the Fab clones followed by ETD allowed a more detailed comparison of the sequences of the Lc and Fd fragments of the shared Fab clones. While the *z-*type fragment ions dominated the mass spectrum, we also detected several *c-*type fragment ions. The masses or *m*/*z*’s of the *c-*type fragment ions of both the Lc and Fd fragments were identical for shared clones generated by either IgdE or BdpK digestion. In contrast, the *z-*type fragment ions of the Fd showed for each fragment ion a mass shift of 101 Da. This observation makes perfect sense, as the distinct digestion sites of the proteases are at the C-terminus of the Fd (Figure 3B).

The differences between the *c-* and *z-*type fragments found for both the Lc and Fd fragments are also reflected in the extracted Pearson correlation (*r*) values when comparing the raw top-down fragmentation data of two “shared” clones. The fragments of the Lc of the same clone digested with the two different proteases shows a reasonable correlation, 0.45 for clone A and 0.33 for clone E, while the Fd shows a much lower correlation, 0.13 for clone A and <0.5 for clone E. Both the values for the Lc and Fd are higher for clone A compared to clone E, which can be explained by the intensities of the clones: while clone A was the highest abundant clone in the profile and showed a rich fragmentation spectrum, clone E was lower abundant, reflected also by a less rich fragmentation spectrum. As a result of this less rich fragmentation spectrum, the *c-*type fragment ions, which were supposed to be similar after both digestions, were almost absent. Correlation analysis of the top-down MS/MS spectra of two different clones (A vs E) proved to always be <0.05 (Figure 3A). In summary, this top-down ETD fragmentation data provides additional confirmation that the “shared” clones observed following IgdE and BdpK digestion, characterized by identical retention time and a mass difference of 101 Da, are indeed identical clones with identical sequences.

## Conclusion

In this study, we directly compared the applicability of the proteases IgdE and BdpK for plasma IgG1 clonal profiling by liquid chromatography coupled to mass spectrometry (LC-MS). Our data indicate that BdpK can digest polyclonal plasma IgG1s specifically and, very crucially, that neither BdpK nor IgdE introduces substantial qualitative or quantitative biases in the detected clonal profiles. This validation reinforces our initial assumption that the Fab clonal profiles generated by both IgdE and BdpK truly reflect the antibody profile in blood. BdpK was found to be more efficient than IgdE in digesting polyclonal plasma IgG1s.

Additionally, when used in combination, both proteases can aid in the *de novo* sequencing of intact Fabs. This combination can distinctively assign top-down fragments to either the Lc or the Fd of the antibody by looking at the mass difference of the alike fragment ions between the different digests. When the fragments show no difference in mass for the C- and N-terminal fragments, they are Lc fragments. Conversely, when a 101 Da difference is observed for the C-terminal fragments, they can be considered to be Fd chain fragments. This assignment of top-down fragments may benefit the *de novo* sequencing of antibody clones, as the assignment to the different chains is often a challenge when performing top down *de novo* sequencing of intact Fabs as both Lc and Fd fragments appear in the same fragmentation spectrum.

## Data Availability

The raw mass spectrometry data have been deposited in the MassIVE repository (https://massive.ucsd.edu/ProteoSAFe/static/massive.jsp) under accession code MSV000092676.

## Supporting Information Available

Experimental details and additional proof for our methods, including LC-MS traces and overlap and intensity analysis after BdpK and IgdE digestion (PDF)
