# Supplementary material for: Direct Comparison of the Hinge-Cleaving Proteases IgdE and BdpK for LC-MS-Based IgG1 Clonal Profiling
Source: Anal Chem. 2023 Dec 18;96(1):23–7. doi: 10.1021/acs.analchem.3c03712 (PMC10782413; doi:10.1021/acs.analchem.3c03712)
Supplement: Supplementary file 1 — ac3c03712_si_001.pdf [file ac3c03712_si_001.pdf]

Supporting information

Direct comparison of the hinge-cleaving proteases IgdE and BdpK for LC-MS based IgG1 clonal profiling

Danique M.H. van Rijswijck[1,2], Albert Bondt[1,2], Naomi de Kat[1,2], Rolf Lood[3] and Albert J.R. Heck[1,2,*]

[1]Biomolecular Mass Spectrometry and Proteomics, Bijvoet Center for Biomolecular Research and Utrecht Institute for Pharmaceutical Sciences, University of Utrecht, Padualaan 8, Utrecht 3584 CH, the Netherlands

[2]Netherlands Proteomics Center, Padualaan 8, Utrecht 3584 CH, the Netherlands

[3]Genovis AB, Scheelevägen 2, 223 63 Lund, Sweden

[*]Corresponding Author: Albert J.R. Heck, E-mail: a.j.r.heck@uu.nl

Table of Content

This supporting information file includes additional information as described in the main manuscript including:

Experimental S1. Extended Supplementary Materials and Methods section

Figure S1. Comparison of reversed phase (RP) Liquid chromatography Mass spectrometry (LC-MS) intensity profiles of Fab fragments generated by digestion the same plasma sample with either BdpK or IgdE.

Figure S2. Comparison of IgG1 clonal repertoires after IgdE and BdpK digestion: overlap and intensity analysis.

Experimental S1

Human subjects

A set of longitudinally acquired EDTA plasma samples from a healthy Caucasian donor was acquired from Precision Med (Solana Beach, CA, US) as part of the 'normal control collections' (protocol number 7005-8200). The EDTA plasma was collected at three different time points from this donor, with 41 and 76 days between the different time points.

LC-MS(/MS)

To analyze the released intact Fab fragments, we employed a reversed-phase liquid chromatography coupled mass spectrometry (LC-MS) and data processing method, as previously described (11). In short, the collected intact Fab molecules were separated by using a Thermo Fisher Scientific Vanquish Flex UHPLC instrument, equipped with a 1x150 mm MAbPac Reversed Phase HPLC Column. During chromatographic separation, both the column pre-heater and the analytical column chamber were heated to 80°C. The LC was directly coupled to an Orbitrap Exploris 480 MS with BioPharma option (Thermo Fisher Scientific, Bremen, Germany) for intact Fab measurements and to an Orbitrap Fusion Lumos (Thermo Fisher Scientific, San Jose, United States) for ETD MS/MS purposes. The Fab samples were separated over a ~60-minute gradient at 150 µL/min flow rate. Gradient elution was achieved using two mobile phases, A (0.1% HCOOH in MilliQ water) and B (0.1% HCOOH in CH3CN) at a starting mixture of 90%A and 10%B, ramping up from 10 to 25% over 1 min, from 25% to 40% over 54 min, and from 40% to 95% over 1 min. MS data were collected with the instrument operating in intact protein and low-pressure mode. Spray voltage was set at 3.5 kV from minute 2 to minute 50 to prevent salts in the sample from entering the MS, ion transfer tube temperature was set at 350 °C, vaporizer temperature at 100°C, sheath gas flow at 15 arbitrary units, auxiliary gas flow at 5 arbitrary units, and source-induced dissociation (SID) at 15 V. Spectra were recorded with a resolution setting of 7500 (@*m/z* 200) in MS1 allowing improved detection of charge distributions of large proteins (>30 kDa). Scans were acquired in the range of 500 to 4000 *m/z* using an automated gain control (AGC) target of 300% and a maximum injection time set to 50 milliseconds. For each scan, 5 micro-scans were recorded. The electron transfer dissociation (ETD) of the Fab fragments was performed using the following settings: 16 milliseconds reaction time, a maximum ETD injection time of 200 milliseconds, and an AGC target of 1e6 for the ETD reagent. For the data-dependent MS/MS acquisition strategy, the intensity threshold was set to 2e5 of minimum precursor intensity. MS/MS scans were recorded in the range of 350 to 5,000 *m/z* using high mass range quadrupole isolation. Reduced Fab samples, containing separate light chains and the N-terminal parts of the heavy chain (Fd) fragments were

analyzed with a resolution setting of 120,000 (@*m/z* 200) in MS1. The precursor ions of interest were mass-selected by the quadrupole in a 4 *m/z* isolation window and accumulated to the AGC target prior to fragmentation. The ETD was performed as shown for the intact Fabs, using a maximum ETD injection time of 200 milliseconds and the AGC target of 1e6 for the ETD reagent. For the data-dependent MS/MS acquisition strategy for the Fab chains we used two scans, we set the intensity threshold to 5e4 of minimum precursor intensity and included charge states between 10 and 50.

Clonal profiling and data analysis

The retention times (RT) and masses (in Dalton) of each of the intact Fab molecules were retrieved from the generated RAW files using BioPharmaFinder 3.2 (Thermo Fisher Scientific). Deconvolution was performed using the ReSpect algorithm (Thermo Fisher Scientific, Bremen, Germany) between 5 and 57 minutes using 0.1 minute sliding windows with 25% offset and a merge tolerance of 30 parts per million (ppm), and noise rejection was set at 95%. The output range was set between 10,000- 100,000 Da with a target mass of 48,000 Da (the average mass of a Fab fragment) and a mass tolerance of 30 ppm. Charge states between 10 and 60 were included, and the Intact Protein Peak model was selected. Further data analysis was performed using in-house Python 3.9.13 scripts using libraries: pandas 1.4.4 , Numpy 1.21.5 (25), Scipy 1.9.1 (26), matplotlib 3.5.2 (27) and seaborn 0.11.2. Masses of the BioPharmaFinder identifications were recalculated using an intensity weighted mean, considering only the most intense peaks comprising 90% of the total intensity. For the intact Fab components between 45,000 and 53,000 Da with the most intense charge state above 1000 *m/z* and BPF score > 40 were considered likely Fab fragments of IgG1 clones. To match the clones between runs, hierarchical clustering was performed using average linkage (UPGMA) L∞ distance. Flat clusters were created with a cophenetic distance constraint derived from the mass and retention time tolerance, defined as 3 times the standard deviation of the mass and RT found for the most intense antibodies shared across the different MS runs (0.5 Dalton and 0.5 minutes, respectively). The ETD fragment ions originating from the intact Fabs or separate Lc and Fd chains were deconvoluted using the Xtract algorithm including charges between 1 and 30, and analyzed accordingly (28).

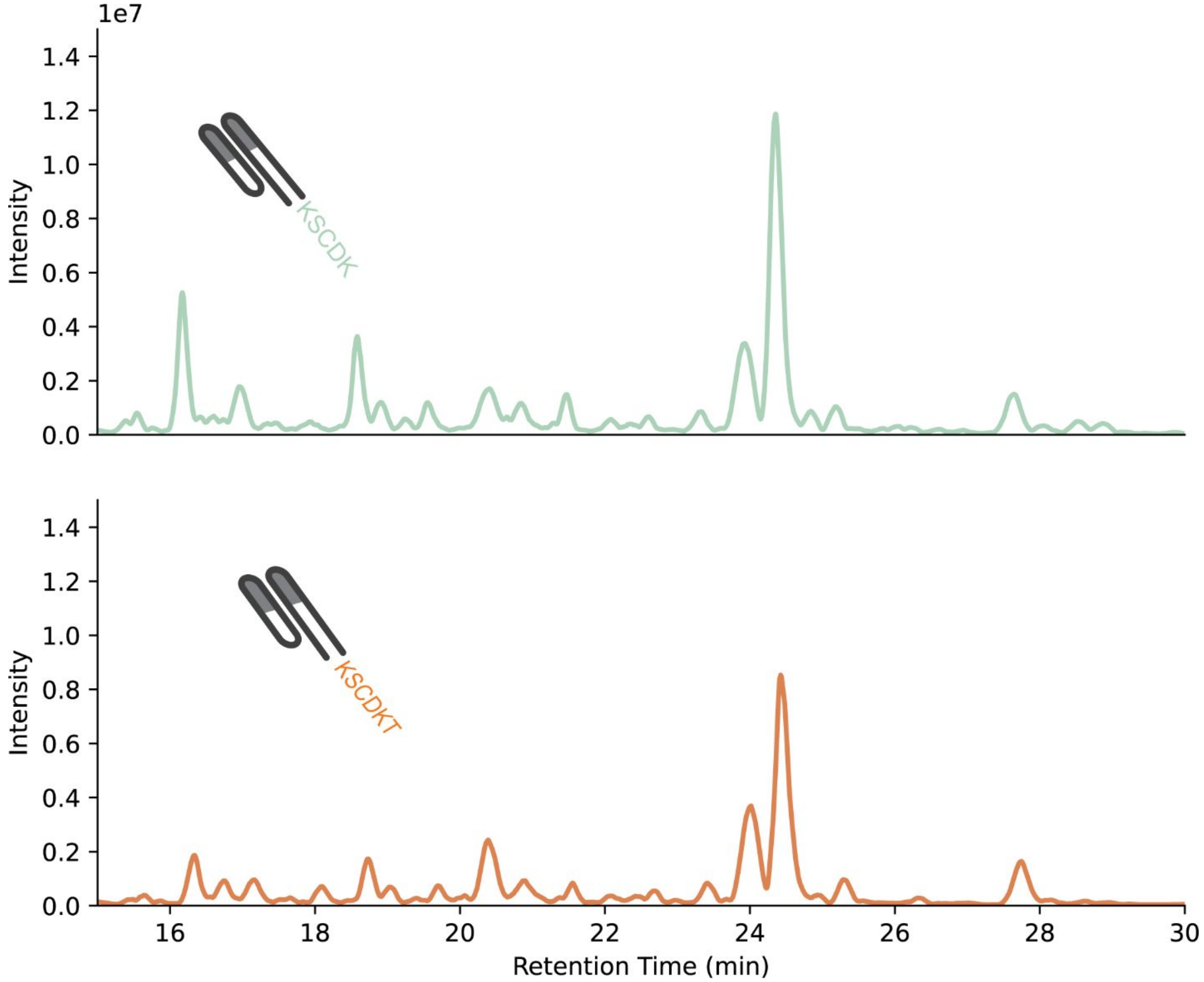

Figure S1. Comparison of reversed phase (RP) Liquid chromatography Mass spectrometry (LC-MS) intensity profiles of Fab fragments generated by digestion the same plasma sample with either BdpK (Top trace) or IgdE (Bottom trace). The x-axis represents the retention time in minutes (min), while the y-axis represents the intensities. Overall, the retention time profiles look very alike between the same sample digested with the two different proteases.

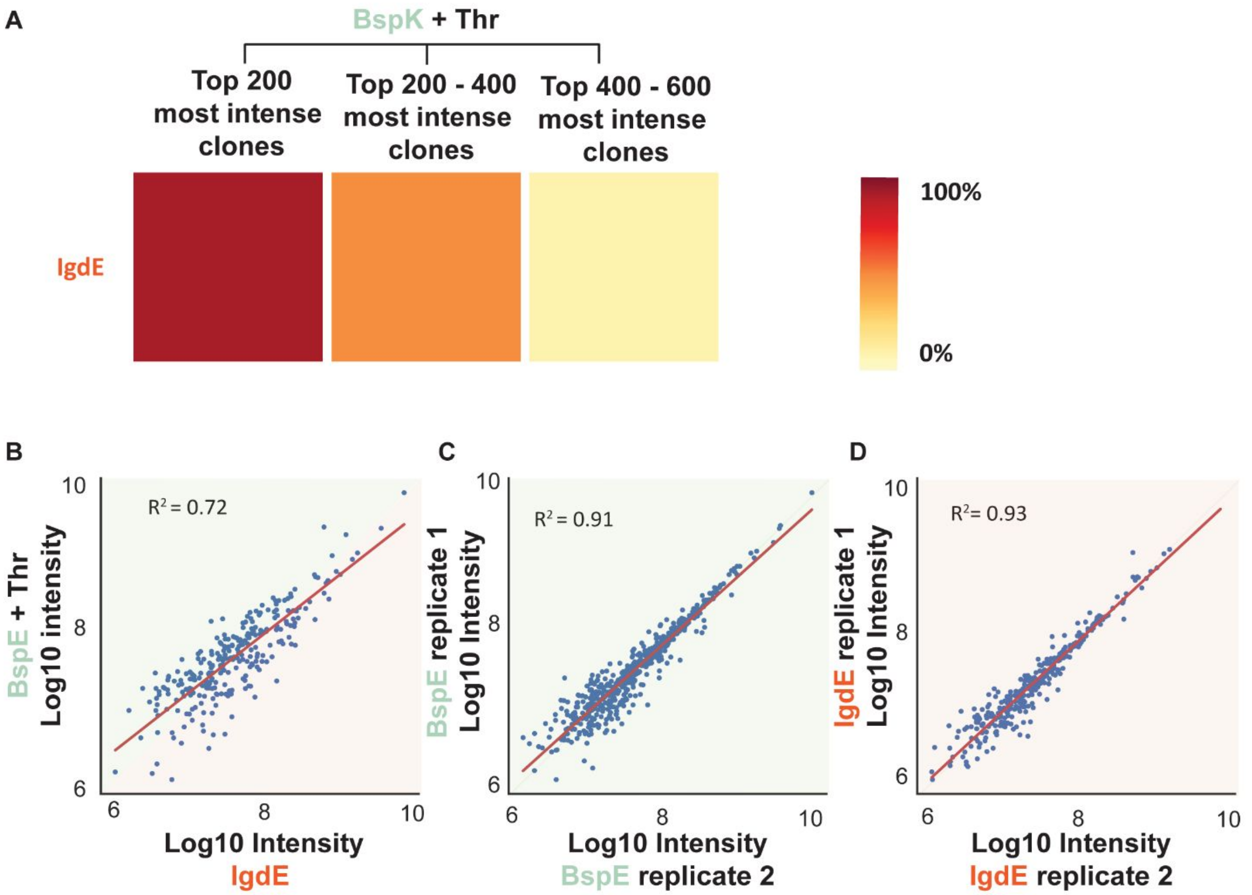

Figure S2. Comparison of IgG1 clonal repertoires after IgdE and BdpK digestion: overlap and intensity analysis. (A) For the same plasma sample, we detected a higher overall number of clones and total ion intensity after digestion with BdpK compared to after digestion with IgdE. We divided the clones that were detected after BdpK digestion in 3 groups based on their intensity: the top 0-200 most intense clones, the in intensity next 200-400 most intense clones, and the in intensity next 400-600 most intense clones. This allowed for a direct comparison with the clonal intensities observed following IgdE digestion, including both the more intense and less intense clones. A mass increment of 101 Dalton was applied to the BdpK digested clones to compensate for the Threonine (Thr) difference, enabling the direct comparison between IgdE and BdpK. The persistent Fab clones, quantified based on intensity, are presented as a percentage using the color bar. The overlap between IgdE and the most intense clones after BdpK digestion (Top 200 most intense clones) was 92%, whereas the overlap with the less abundant clones after BdpK digestion (the in intensity ranked 400-600 most intense clones) was only 9%. (B) Scatterplot comparing the Log10 intensity of individual clones which were detected after both IgdE (x-axis) and BdpK (y-axis) digestion. Each dot represents a unique clone which was detected after both digestions. A trendline (in red) was fitted through all the dots suggests a slightly higher intensity for the clones found after BdpK digestion and reveals on average a good correlation with a R^2 of 0.72. (C) Scatter plot comparing the Log10 intensities of individual clones detected in two technical replicates of BdpK digestion on the same plasma sample. Each dot represents a unique clone, and the trendline fitted using all the dots shows a tendency to through 0 and shows a R^2 of 0.91 (in red). (D) Scatter plot comparing the Log10 intensities of individual clones detected in two technical replicates of IgdE digestion on the same plasma sample. Each dot represents a unique clone, and the trendline fitted using all the dots shows a tendency to go through 0 and a R^2 of 0.92(in red).
